# Host Genetic Liability for Severe COVID-19 Associates with Alcohol Drinking Behavior and Diabetic Outcomes in Participants of European Descent

**DOI:** 10.3389/fgene.2021.765247

**Published:** 2021-12-13

**Authors:** Frank R Wendt, Antonella De Lillo, Gita A Pathak, Flavio De Angelis, COVID-19 Host Genetics Initiative, Renato Polimanti

**Affiliations:** ^1^ Division of Human Genetics in Psychiatry, Yale School of Medicine, New Haven, CT, United States; ^2^ VA CT Healthcare System, West Haven, CT, United States

**Keywords:** alcohol, causal inference, COVID-19, diabetes, genetic overlap, SARS-CoV-2

## Abstract

Risk factors and long-term consequences of COVID-19 infection are unclear but can be investigated with large-scale genomic data. To distinguish correlation from causation, we performed *in-silico* analyses of three COVID-19 outcomes (N > 1,000,000). We show genetic correlation and putative causality with depressive symptoms, metformin use (genetic causality proportion (gĉp) with severe respiratory COVID-19 = 0.576, *p* = 1.07 × 10^−5^ and hospitalized *COVID-19* = 0.713, *p* = 0.003), and alcohol drinking status (gĉp with severe respiratory *COVID-19* = 0.633, *p* = 7.04 × 10^−5^ and hospitalized COVID-19 = 0.848, *p* = 4.13 × 10^−13^). COVID-19 risk loci associated with several hematologic biomarkers. Comprehensive findings inform genetic contributions to COVID-19 epidemiology, molecular mechanisms, and risk factors and potential long-term health effects of severe response to infection.

## Introduction

Host genetic liability to severe COVID-19 (coronavirus disease 2019) following SARS-Cov-2 (severe acute respiratory syndrome coronavirus 2) infection is of immediate clinical interest. (Ji et al., 2020). While preexisting comorbidities, including hypertension, type 2 diabetes, and asthma, have been characterized in large population cohorts, it remains unclear which long-term health consequence may arise following COVID-19 infection. (Atkins et al., 2020). Furthermore, the direction of epidemiological observations is confounded by many external factors such that bidirectional effects have been reported for comorbidities such as type 2 diabetes. (Atkins et al., 2020; Rubino et al., 2020).

To understand better the association of biological measurements, lifestyle indicators, biomarkers, and health and medical records with COVID-19 susceptibility, we performed analyses to distinguish genetic correlation from genetically informed causal effects using large-scale genomic data of COVID-19 outcome severity in over 1,000,000 participants from the COVID-19 Host Genetics Initiative.

## Materials and Methods

### Genome-Wide Association Studies

Genome-wide association statistics were accessed from the COVID-19 Host Genetics Initiative (HGI; https://www.covid19hg.org/; September 30, 2020 release date) (COVID-19 Host Genetics Initiative, 2020) for six COVID-19 phenotypes (Supplementary Table S1): (i) *very severe respiratory confirmed COVID-19 versus population* (COVID_19_A2; N_case_ = 2,972, N_control_ = 284,472), (ii) *hospitalized COVID-19 versus not hospitalized COVID-19*, (iii) *hospitalized COVID-19 versus population* (COVID_19_B2; N_case_ = 6,942, N_control_ = 1,012,809), (iv) *COVID-19 versus lab/self-reported negatives*, (v) *COVID-19 versus population* (COVID_19_C2; N_case_ = 17,607, N_control_ = 1,345,334), and (vi) *predicted COVID-19 from self-reported symptoms versus predicted or self-reported non-COVID-19*. (COVID-19 Host Genetics Initiative, 2021). Note that traits B2 and C2 were restricted to European ancestry participants only, excluding contributions from 23andMe and all other population groups. Trait A2 was only available as a meta-analysis of several ancestry groups with European ancestry samples comprising over 99% of the GWAS meta-analysis.

### Linkage Disequilibrium Score Regression

Using Linkage Disequilibrium Score Regression (LDSC (Bulik-Sullivan et al., 2015a)), we calculated common variant heritability (h^2^) for each COVID-19 outcome. Three outcomes presented with SNP-based h^2^ significantly different from zero: *very severe respiratory confirmed COVID-19 versus population*, *hospitalized COVID-19 versus population*, and *COVID-19 versus population*. The h^2^ estimates of three other COVID-19 outcomes curated by HGI were not significantly different from 0 (Supplementary Table S1).

For two COVID-19 phenotypes with h^2^ z-scores > 4 (the threshold recommended by LDSC developers), (Bulik-Sullivan et al., 2015b), severe respiratory COVID-19 and hospitalized COVID-19, we then estimated their genetic correlation (r_g_) with 4,083 phenotypes from the UK Biobank (UKB, see http://www.nealelab.is/uk-biobank). LDSC analyses were based on linkage disequilibrium information from the 1,000 Genomes Project (1kGP) European reference population. When available for continuous traits, we restricted our analyses to genome-wide association statistics generated from inverse-rank normalized phenotypes. Multiple testing correction was applied to genetic correlation results using the false discovery method (FDR q < 0.05) based on the number of COVID-19 outcomes (two traits with h^2^ z-score > 4) and the number of suitably powered UK Biobank phenotypes (772 traits with h^2^ z-scores > 4) against which r_g_ was tested.

### Latent Causal Variable Analysis

To distinguish between genetic correlation and causative effects, we applied the Latent Causal Variable (LCV) approach to all nominally significant genetic correlations. (O'Connor and Price, 2018). Under the assumption of a single effect-size distribution in per-trait GWAS, LCV tests for the presence of a single latent trait connecting COVID-19 outcomes to UKB phenotypes. LCV was performed in R using the 1kGP European reference LD panel and genome-wide association statistics for SNPs with minor allele frequencies >5%. Variants in the major histocompatibility complex region of the genome were excluded because of its complex LD structure. LCV genetic causality proportion (gĉp) estimates were only interpreted for trait pairs where both traits exhibit LCV-calculated h^2^ z-scores ≥ 7, as recommended by the LCV developers. (O'Connor and Price, 2018). The gĉp estimate ranges from 0 to 1 with values near zero indicating partial causality and values approaching 1 indicating full causality. The sign of the gĉp indicates the direction of the causal relationship. In this study, positive gĉp indicates that the COVID-19 outcome causes the second phenotype while negative gĉp indicates that the second phenotype causes the COVID-19 outcome. LCV developers indicated that gĉp >0.7 can be interpreted as evidence of a strong causal relationship between trait pairs. (O'Connor and Price, 2018). Multiple testing correction was applied using the FDR method (q < 0.05) based on the number of COVID-19 outcomes (N = 2) and the number of suitably powered UK Biobank phenotypes (N = 188 traits with h^2^ z-scores ≥ 7) against which r_g_ also was tested.

### Replication

Significant genetic correlations and latent causal effects were replicated using the FinnGen resource (release 5; accessed September 2021). A total of 20 traits could be mapped to FinnGen for replication.

### Phenome-wide Association in Pan-UKB

Phenome-wide association studies (PheWAS) were performed for 14 loci associated with one of the three heritable COVID-19 outcomes (Supplementary Table S2) using the Pan-ancestry UK Biobank resource (available at https://pan.ukbb.broadinstitute.org/downloads). We analyzed genome-wide association statistics generated from the analysis of 7,218 phenotypes in six ancestries: European (N = 420,531), Central/South Asian (N = 8,876), African (N = 6,636), East Asian (N = 2,709), Middle Eastern (N = 1,599), and Admixed American (N = 980). Pan-UKB traits were analyzed if they had 100 cases in European ancestry or 50 cases in all other ancestries. Association statistics were covaried with sex, age, age^2^, sex×age, sex×age^2^, and the first ten within-ancestry principal components. A detailed description of the methods used to generate these data is available at https://pan.ukbb.broadinstitute.org/. Multiple testing correction was performed for the number of phenotypes (N = 7,218) and ancestry groups (N = 6) using the p.adjust (method = “fdr”) function of R.

### Statistical Comparison of Effect Estimates

To compare the magnitude of genetic correlation and genetical causality proportion between COVID-19 outcomes, we performed two-sided Z-tests. P-values for each Z-test were corrected for multiple testing using the false discovery method.

## Results and DISCUSSION

### Genetic Correlation and SNP-Heritability

Genome-wide association statistics were accessed from the COVID-19 Host Genetics Initiative (September 2020; see Methods). (COVID-19 Host Genetics Initiative, 2020). Assuming a population prevalence of 1%, we tested the SNP-h^2^ of the traits (i) *severe respiratory COVID-19* (h^2^ = 0.129, *p* = 5.95 × 10^−8^), (ii) *hospitalized COVID-19* (h^2^ = 0.0411, *p* = 2.74 × 10^−5^), and (iii) *COVID-19 versus population* (h^2^ = 0.009, *p* = 0.007). All three trait SNP-h^2^ estimates were significantly different from zero, indicating that the variability in each phenotype is at least partially explained by GWAS SNPs (Supplementary Tables S1–S2). LDSC developers recommend that r_g_ be estimated with traits whose h^2^ z-score > 4, permitting tests of r_g_ for *severe respiratory COVID-19* (h^2^ z-score = 5.38) and *hospitalized COVID-19* (h^2^ z-score = 4.11).

**FIGURE 1:**
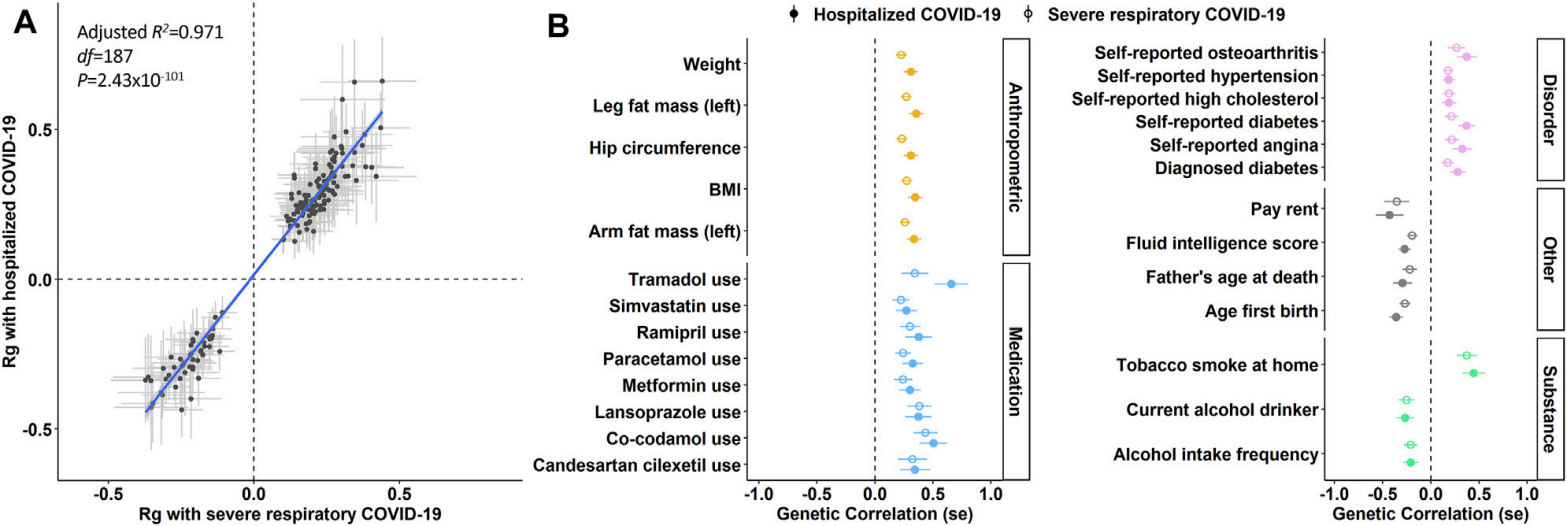
Pleiotropy of genetic risk for COVID-19 susceptibility. **(A)** all 188 traits with nominally significant genetic correlation (Rg) with severe respiratory COVID-19 and hospitalized COVID-19. Each data point represents a single trait and error bars around each data point represent the standard error of the Rg point estimate. The blue line represents a linear model of Rg magnitude weighted by the standard error of each point estimate. **(B)** a subset of traits associated with severe respiratory COVID-19 (open circles) and hospitalized COVID-19 (solid circles) after multiple testing correction (total = 111 traits with FDR q < 0.05; Supplementary Table S3).

We next tested for pleiotropy between COVID-19 risk loci and 4,083 phenotypes from the UK Biobank using the LDSC method. *Severe respiratory COVID-19* and *hospitalized COVID-19* were genetically correlated with 127 and 174 phenotypes, respectively (Figure 1; Supplementary Table S3), reflecting 188 traits. A total of 111/184 phenotypes were genetically correlated with both COVID-19 outcomes (FDR q < 0.05) and there were no differences in r_g_ magnitude between each phenotype and the COVID-19 outcomes. The most significant genetic correlate of COVID-19 outcomes was *waist circumference* (*severe respiratory COVID-19* r_g_ = 0.272, *p* = 2.18 × 10^−9^ and *hospitalized COVID-19* r_g_ = 0.342, *p* = 1.66 × 10^−8^).

### Cross-Trait Latent Causal Effects

**FIGURE 2:**
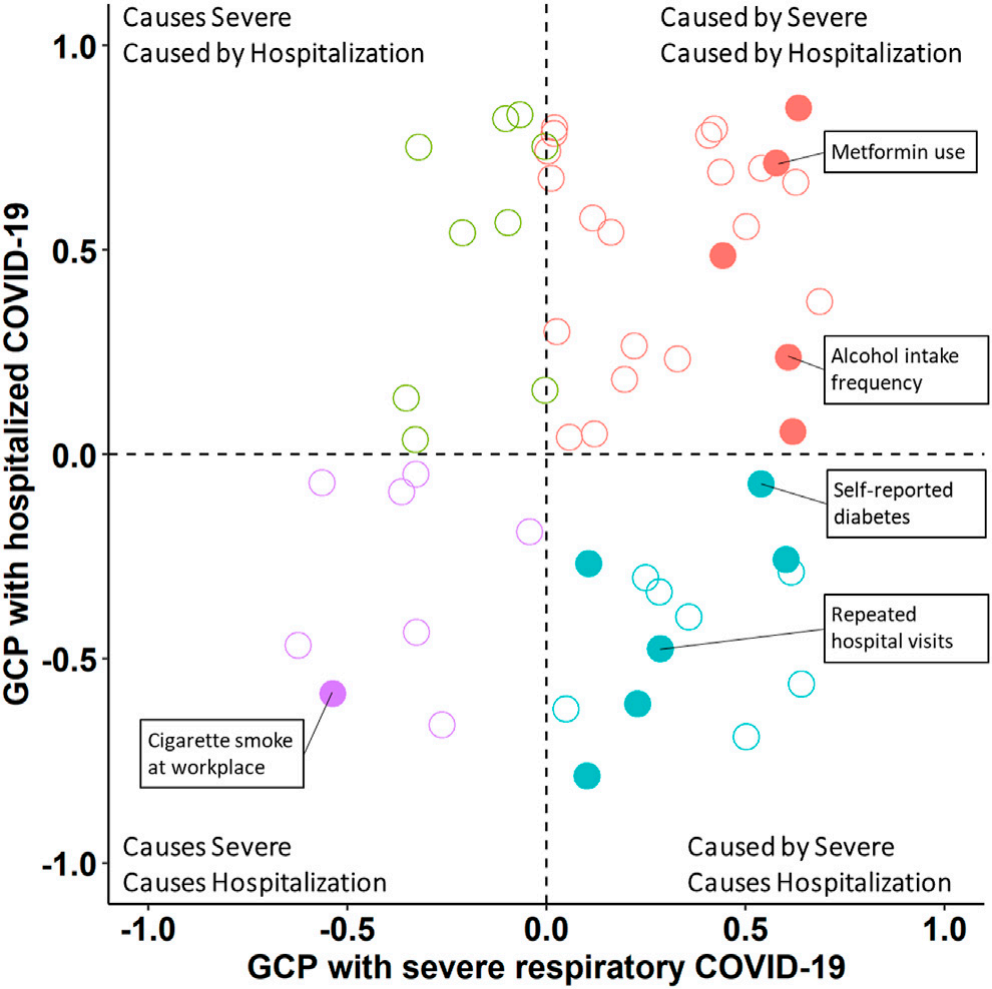
Latent causal relationships between COVID-19 outcomes and significantly genetically correlated traits. Solid circles indicate a trait with significant genetic causality proportion (gcp) with respect to severe respiratory COVID-19 and hospitalized COVID-19 and empty circles indicate non-significant gcp estimates. The color of each data point corresponds to the pattern of causal effect detected (labeled in the corner of each quadrant). Select traits are labeled in each quadrant and all gcp estimates are provided in Supplementary Table S4.

To distinguish between genetic correlation and causal effects, we applied the LCV method. (O'Connor and Price, 2018). LCV tests performed against 188 genetic correlates (Supplementary Table S3) revealed 24 and 42 putative causal relationships with *severe respiratory COVID-19* and *hospitalized COVID-19*, respectively (Figure 2; Supplementary Table S4). One trait, cigarette smoke at workplace, had a causal effect on both *severe respiratory COVID-19* (gĉp = 0.537, *p* = 3.81 × 10^−5^) and *hospitalized COVID-19* (gĉp = 0.585, *p* = 3.52 × 10^−7^). COVID-19 outcomes both had causal effects on several traits including frequency of alcohol intake (*severe respiratory COVID-19* gĉp = 0.608, p = 4.24 × 10^−16^ and *hospitalized COVID-19* gĉp = 0.320, p = 5.85 × 10^−13^) and inability to stop worrying (*severe respiratory COVID-19* gĉp = 0.619, *p* = 3.10 × 10^−9^ and *hospitalized COVID-19* gĉp = 0.213, p = 1.43 × 10^−4^). Finally, several traits were caused by *severe respiratory COVID-19* and causal for *hospitalized COVID-19*: self-reported diabetes (*severe respiratory COVID-19* gĉp = 0.539, p = 1.42 × 10^−12^ and *hospitalized COVID-19* gĉp = 0.072, p = 1.02 × 10^−5^) and adopted as a child. Based on pairwise Z-tests, were no significant differences between gĉp estimates between COVID-19 outcome definitions.

### Replication of r_g_ and LCV in FinnGen

In FinnGen release 5, we mapped 188 significant genetic correlates to 18 phenotypes. Note that 1-to-1 trait matching was not possible for most traits. For example, anthropometric measurements make up 22% of the significant genetic correlates but they are not available in FinnGen. Genetic correlation between two COVID-19 outcomes and 18 FinnGen traits identified seven replicated pleiotropic effects (Supplementary Table S3). All replicated genetic correlations were in the same direction as the discovery r_g_ in UKB and there were no differences in magnitude when comparing UKB and FinnGen. We replicated (*p* < 0.05) the genetic correlation between *hospitalized COVID-19* and COPD, depression, diabetes, pain, tramadol use, and paracetamol use. We replicated a single genetic correlation with *severe respiratory COVID-19* compared to hypertension. Of these seven traits, three had a significant gĉp with either *severe respiratory COVID-19* or *hospitalized COVID-19* in the discovery phase with UKB: pain, diagnosed diabetes, and current depression.

### Phenome-wide Association Study of COVID-19 Associated Loci

**FIGURE 3:**
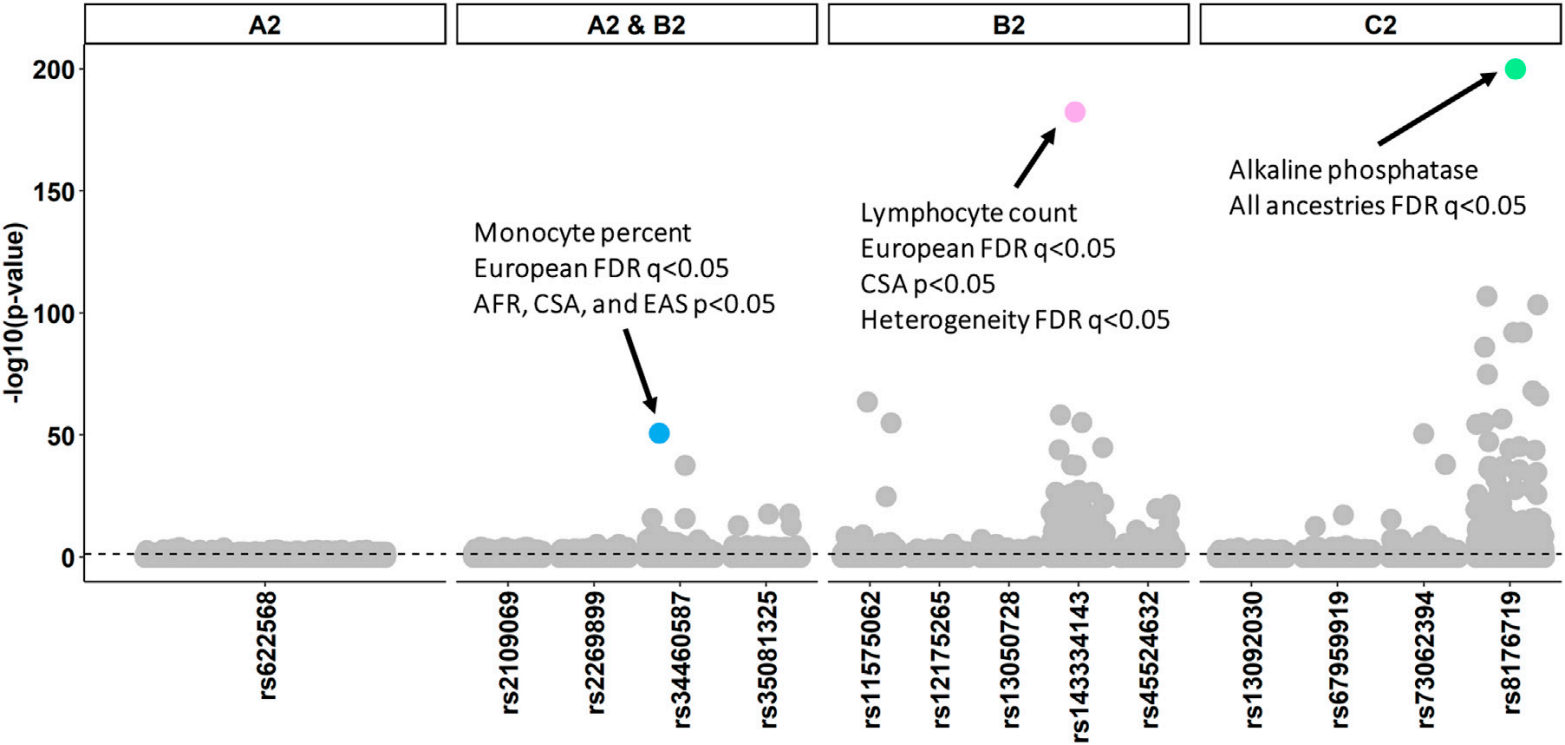
Phenome-wide association study (PheWAS) of risk loci from three COVID-19 outcomes: A2: very severe respiratory confirmed COVID-19 versus population, B2: hospitalized COVID-19 versus population, and C2: COVID-19 versus population. Each facet details the pleiotropic effects of loci detected by GWAS of the indicated COVID-19 outcome. Each data point corresponds to a single trait assessed in UK Biobank participants of European descent. For the top associations of interest, the association between SNP and phenotypes across all ancestries is described. Details of the effect of each SNP in six populations from the Pan-ancestry UKB are provided in Supplementary Table S5.

To test the effects of COVID-19 liability loci on other features of the human phenome, we analyzed genome-wide association statistics from 7,218 phenotypes assessed in European (N = 420,531), Central/South Asian (N = 8,876), African (N = 6,636), East Asian (N = 2,709), Middle Eastern (N = 1,599), and Admixed American (N = 980) ancestries from the Pan-ancestry UKB analysis. Because all three COVID-19 outcomes with significant SNP-h^2^ harbor genome-wide significant loci, we include all of these SNPs in our PheWAS. The 14 COVID-19 liability loci across three severity outcomes were associated with 439 phenotypes (FDR q < 0.05, Figure 3; Supplementary Table S5). Alkaline phosphatase was negatively associated with rs8176719 (*ABO* locus) in all six ancestries. The *ABO* locus exhibited effect size heterogeneity (Supplementary Figure S1) with respect to low-density lipoprotein cholesterol concentration (cross-ancestry meta-analysis β = 0.057, *p* = 3.01 × 10^−5^) and hemoglobin concentration (cross-ancestry meta-analysis β = 0.040, *p* = 1.67 × 10^−5^). We also detected effect size heterogeneity at rs143334143 (*CCHCR1*, lymphocyte count meta-analysis β = 0.144, *p* = 5.16 × 10^−179^, p_het_ = 2.95 × 10^−5^) and rs45524632 (*KEAP1*, heel bone mineral density T-score meta-analysis β = -0.042, *p* = 2.59 × 10^−5^, p_het_<2.95 × 10^−5^).

## Discussion

In light of the 2020 COVID-19 pandemic and ongoing 2021 interpersonal distancing protocols, host genetic susceptibility to severe responses to SARS-CoV-2 infection is critical. We used genome-wide data to uncover overlap and putative causal relationships between genetic liability to COVID-19 severity, preclinical risk factors (e.g., alcohol consumption), (Ko et al., 2020), and long-term consequences of infection (e.g., diabetes). (Del Rio et al., 2020). Our most notable findings reflect (1) causal consequences of cigarette smoke exposure and alcohol consumption on COVID-19, (2) causal consequences of COVID-19 on diabetes, and (3) ABO blood type effects on COVID-19 severity across ancestry. (Ellinghaus et al., 2020).

Exposure to cigarette smoke and tobacco products through either direct consumption or as an environmental exposure in community spaces has garnered considerable attention. On one hand, direct consumption of tobacco via smoking was associated with a significantly worse COVID-19 prognosis than non-smokers. (Peng et al., 2021). Smoking may cause a greater abundance of respiratory epithelial angiotensin-converting enzyme-2 (ACE2) receptors contributing to greater viral load as ACE2 is the major binding point for SARS-CoV-2 via spike protein interaction. (Brake et al., 2020; Leung et al., 2020). Furthermore, as SARS-CoV-2 is transmitted via aerosolized salivary particles, environmental exposure to the virus may contribute to higher transmission. (Ahmed et al., 2020).

The relationships between alcohol and diabetes with COVID-19 severity demonstrate that epidemiologic observations between them are due, in part, to putative causal effects. (Saengow et al., 2020). Persons with diabetes have been identified as some of the most high-risk individuals for COVID-19 and there are several instances of spontaneous diabetes onset following COVID-19 recovery. (Chee et al., 2020). As risk factors, diabetes and alcohol consumption accentuate two prominent mechanistic hypotheses leading to severe COVID-19 and higher mortality: irregular blood viscosity and hyperglycemia. Increased glucose levels directly increase SARS-CoV-2 replication and proliferation. (Lim et al., 2021). This relationship is so pronounced that antidiabetic medications and other glucose-lowering medications may be viable treatments to reduce mortality in COVID-19 positive diabetics. (Luo et al., 2020; Sardu et al., 2020).

In line with other studies, (Hawkins et al., 2020; Mena et al., 2021), we hypothesize that socioeconomic status likely mediates many of the effects observed here, such as those between COVID-19 outcomes and *adopted as a child*, home locations, and workplace conditions. For example, in our previous work, we demonstrated that regions of the genome associated with household income attenuated the effect of body mass index on *severe respiratory COVID-19*. (Cabrera-Mendoza et al., 2021). The genes associated with household income have been linked to brain regions associated with educational attainment such as the anterior cingulate cortex and cerebellum. Furthermore, medium spiny neurons and serotonergic neurons in these regions appear to play a role in higher household income. (Hill et al., 2019a). Therefore, genetic studies of socioeconomic variables may capture genetic and environmental contributions to brain structure and function that may be relevant for other social factors associated with health, such as BMI or the childhood adversity and poor work condition information detected here. (Hill et al., 2019b; Polimanti et al., 2019). The LCV method used here is not suited for multivariable analyses and future work is necessary to untangle which of the detected effects are independent of various measures of socioeconomic status. (O'Connor and Price, 2018).

With single-SNP measures we recapitulate the relationship between COVID-19 severity and diabetes outcomes by detecting consistent negative association between rs8176719 (*ABO* locus) and alkaline phosphatase, an enzyme with evidence of protective effects against diabetes when present in sufficient concentrations. (Malo, 2015).

Our findings have two primary limitations. First, since we investigated datasets generated from participants of European descent, our findings may not translate to other ancestries. Second, the methods used herein fail to entertain multivariable latent causal factors, such as socioeconomic status, which has a documented history of confounding causal inference studies and disproportionately influencing COVID-19 infection. (Hawkins et al., 2020; Muniz Carvalho et al., 2020; Mena et al., 2021). These findings reflect potential measures to refine, and/or improve accuracy and generalizability of COVID-19 severity outcomes with epidemiological and self-report information. (Ji et al., 2020). The detected risk factors and potential chronic outcomes have critical public health consequences on the long-term economic burden of the COVID-19 pandemic (Miller et al., 2020).

## Data Availability

The original contributions presented in the study are included in the article/Supplementary Material, further inquiries can be directed to the corresponding author.

## Supplementary Material

Supplementary Figure S1 Heterogeneous effects of rs143334143 on lymphocyte count and rs45524632 on heel bone T-score across six world populations from the Pan-UK Biobank. Double asterisks indicate significance after multiple testing correction (FDR 5%) while single asterisks indicate nominal significance (p<0.05). Ancestry assignments are: AFR (African), AMR (admixed American), CSA (Central/South Asian), EAS (East Asian), EUR (European), MID (Middle Eastern), Meta (trans-ancestry meta-analysis). MAF denotes the SNP minor allele frequency.
